# Network-level pavement maintenance and rehabilitation decision-making with the optimized annual budget

**DOI:** 10.1371/journal.pone.0287426

**Published:** 2023-10-26

**Authors:** Lin Chen

**Affiliations:** 1 College of Transportation Engineering, Chang’an University, Xi’an, China; 2 Engineering Research Center of Road Transportation Decarbonization, Ministry of Education, Xi’an, China; Southwest Jiaotong University, CHINA

## Abstract

Maintenance and rehabilitation (M&R) is necessary to keep pavement networks in good condition. Due to the capital intensity, M&R funding is always insufficient. The annual budget, determining the available funding, is a critical criterion when planning M&R treatments. However, its values are often given, and the determination of the values is seldom discussed. To fill the gap, this paper focuses on both the determination of annual budgets and the budget allocations, and therefore enhances the network-level decision-making on M&R by developing a Multi-Objective Optimization (MOO) method. This method does not only optimize and trade off the annual budgets and their consequences, but also allocates the funding across the entire network through generating the optimized M&R decisions. According to a case study with 50 segments, the developed method successfully and effectively identified non-linear discrete relationship between the minimized annual budgets and the maximized M&R benefits subject to all the constraints, and generated the optimized annual budget allocation for each M&R decision. The achievements of this paper can be used to enhance the efficiency of M&R decisions and contribute to informed pavement management.

## Introduction

Pavement deteriorates over time as a result of usage and climate. Hence routine and periodic maintenance and rehabilitation (M&R) is necessary to keep the pavement in an acceptable physical condition and functioning properly against deterioration and damages [1]. However, pavement infrastructures are capital-intensive, and their M&R is very costly. Underfunding and the continuous pavement deterioration have been the major challenge for pavement management departments all over the world. Furthermore, due to the economic depression and the common pressure for saving public financial means, the pavement M&R is facing severer challenges of insufficient funding now than ever. In United States of America (USA), the pavement infrastructures in “poor” condition have increased by more than 2% over the past decade, and the funding gap for M&R grows to $435 billion [2]. Hence, an efficient amount of M&R funding is increasingly important when managing pavement.

A M&R annual budget, determining the available annual funding for M&R treatments, can save significant capital expenditure and yield the best return on M&R investment [3]. Generally, a higher annual budget results in a better pavement network, whilst their accurate relationship is unclear. A high annual budget may not be efficient considering the strained finances; and a low annual budget causes the delays on M&R treatments, which contributes to the accumulation of yearly M&R backlogs and accelerates the road deterioration [4, 5]. According to Abukhalil and Smadi, the scarcity of funding on M&R backlog had increased from $549.5 billion in 2009 to $836 billion in 2017 [6]. Therefore, an appropriate annual budget should be sufficient to allow “the right treatment on the right pavement at the right time” [7] and result in the highest investment return.

Although the annual budget of M&R is important, its determination is mostly experience-oriented. In the industry, often the annual budget is artificially decided in competition with other budget allocations by the treasury. This indicates that decision makers are constrained by the funding across the entire network subject to the annual budget [8]. However, the efficiency of the artificially decided values is unknown, and the decision makers are not informed with other choices of annual budgets.

In academic research, most M&R decisions consider annual budgets when planning the M&R treatments for their networks [9–12]. They primarily focus on the budget allocation with the given budget values, whilst the determination of the budget values and the budget efficiency are rarely discussed. They are inadequate to identify an efficient M&R annual budget for pavement networks.

Therefore, in both industry and academic research, assistance is needed to determine the appropriate annual budget of M&R when maintaining and rehabilitating a pavement network. To fill the need, this paper develops a method that does not only design M&R treatments but also presents the optimized choices of the annual budgets and their consequences for the network-level pavement M&R planning. The developed method can assist decision makers in determining the appropriate values of annual budgets and allocating the budgets across the entire network for pavement M&R and therefore improving the economic efficiency of pavement management.

The following sections of this paper are organized as follows: Section II introduces the background of network-level M&R decision and existing researches. Thereafter, in Section III, a decision-making method is developed to optimize and trade off the annual budgets and their consequences when planning M&R treatments for pavement networks. In Section IV, the developed method is tested with a real pavement network in Texas. Finally, this paper is concluded in Section V.

## Background and literature review

**Fig 1:**
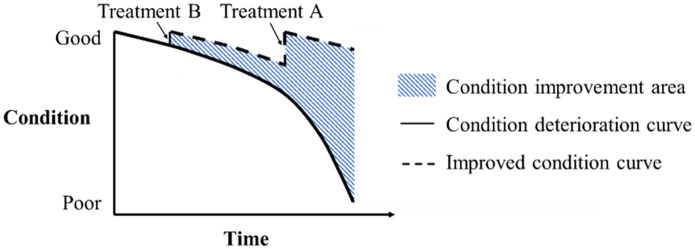
Example of pavement condition deterioration.

M&R decision-making is an essential aspect of network-level Transportation Infrastructure Asset Management [1]. It decides the M&R treatments assigned to the segments of a pavement network across the analysis horizontal [13]. Once constructed, pavement deteriorates until a M&R treatment is applied. As presented in Fig 1, when designing different M&R treatments for a pavement segment at various times, the outcomes, for example, M&R costs, benefits, and pavement condition, are also different.

As early as 1975, Abelson and Flowerdew found the cheapest M&R plan using dynamic programming [14]. Traditional pavement management mainly focuses only on pavement condition and M&R costs, for example, priority treating the worst pavement segments subject to available funding or reducing M&R cost to achieve an acceptable network [15–17]. However, this is not good enough for advance M&R decision [18].

Advance M&R decision should consider various decision criteria, including M&R benefits, pavement condition, funding efficiency, etc., when planning M&R treatments [19–21]. As an important criterion, the annual budget determines the available funding for M&R treatments of the entire pavement network in every year. Based on the statistical analysis, the budget has strong influence on the pavement condition and economical efficiency [22]. The excess funding may not generate increased investment return, while the insufficient funding causes the delays in M&R projects and consequently accelerates the pavement deterioration. According to Galehouse, Moulthrop, and Hicks, if $1 is lacking or improperly spent on M&R, $6-$10 loss would be incurred in later service life of pavement [7]. Hence, an appropriate budget level is important when planning M&R treatments.

Due to the importance, budgets are widely involved in M&R decision-making process [23–26]. However, their values were often artificially given based on experience, and the corresponding problems arise when allocating the assigned funding rather than explaining how to determine the appropriate values of annual budgets [27–29]. Some researchers defined several budget scenarios and generated a M&R strategy or strategies subject to each scenario [30–34]. They presented multiple funding allocation considering the budget scenarios. However, a few specified scenarios were not necessarily sufficient to achieve the full variation of the annual budgets, and the information to define the scenarios is hardly provided.

To understand the effects of annual budgets, Saitoh and Fukuda defined a set of fuzzy constraints to release the budget requirement when planning M&R treatments [35]. Similarly, Liu et al. developed a model for the budget adjustment purpose also by relaxing the actual budget [36]. Their research allowed a better M&R decision even when the decision did not strictly satisfy the budget constraints. Saha and other researchers conducted the sensitivity analysis to clarify how the changing budget affected M&R outcomes, and accordingly generated an optimum annual budget [37, 38]. Shiboub and Assaf changed the M&R budget from $4 million to $7 million for their pavement network, and optimized the budget allocation under each budget scenarios [4]. These studies certified the heavy impacts of annual budgets on M&R decisions, while they were still based on the pre-determined given values of annual budgets, while the information to determine the budget values and the alternative choices of annual budgets were not provided.

When discussing M&R decision-making, Multi-Objective Optimization (MOO) is commonly used, which can help understanding the relationship of decision-making criteria and balancing M&R outcomes. For example, Bai et al. used NSGA II (Nondominated Sorting Genetic Algorithm II) to optimize the performance measures and trade off their variation in the M&R decision-making process [39]. France-Mensah and O’Brien optimized M&R strategies with minimized greenhouse gas emissions, maximized pavement condition improvement and minimized user costs using a mixed integer program and reached a decision with trade-off [40]. Khiavi and Mohammadi traded off the agency cost, the user cost, and the road residual value with NSGA II, and obtained optimal weights of the M&R outcomes for a pavement network in Texas [41]. Other studies also successfully used MOO to generate and trade off the best M&R outcomes, and reached a compromised M&R decision [42–44]. This methodology can also be used to determine the appropriate annual budgets when making M&R decisions.

As discussed above, a large number of studies involved the annual budgets in their decision-making process, even some large networks were analyzed. However, they often focus on the budget allocation, where the budget were factored as constraints. Yet the budget values were given without explaining how to determine the given values. Some researchers discussed the annual budgets using fuzzy constraints, multiple scenarios, or sensitivity analysis in the context of M&R decisions. However, they did not present the alternative choices of the best annual budgets and its consequences. Thus, they were not adequate to benchmark an appropriate annual budget. MOO and trade-offs can help understanding and balancing the optimal variation of outcomes. Therefore, this paper proposes a method that does not only determine appropriate values of annual budgets but also allocates the budgets across the entire network with MOO and trade-offs by generating M&R decisions.

## Decision-making with the optimized annual budget

### MOO application in M&R decision-making

**Fig 2:**
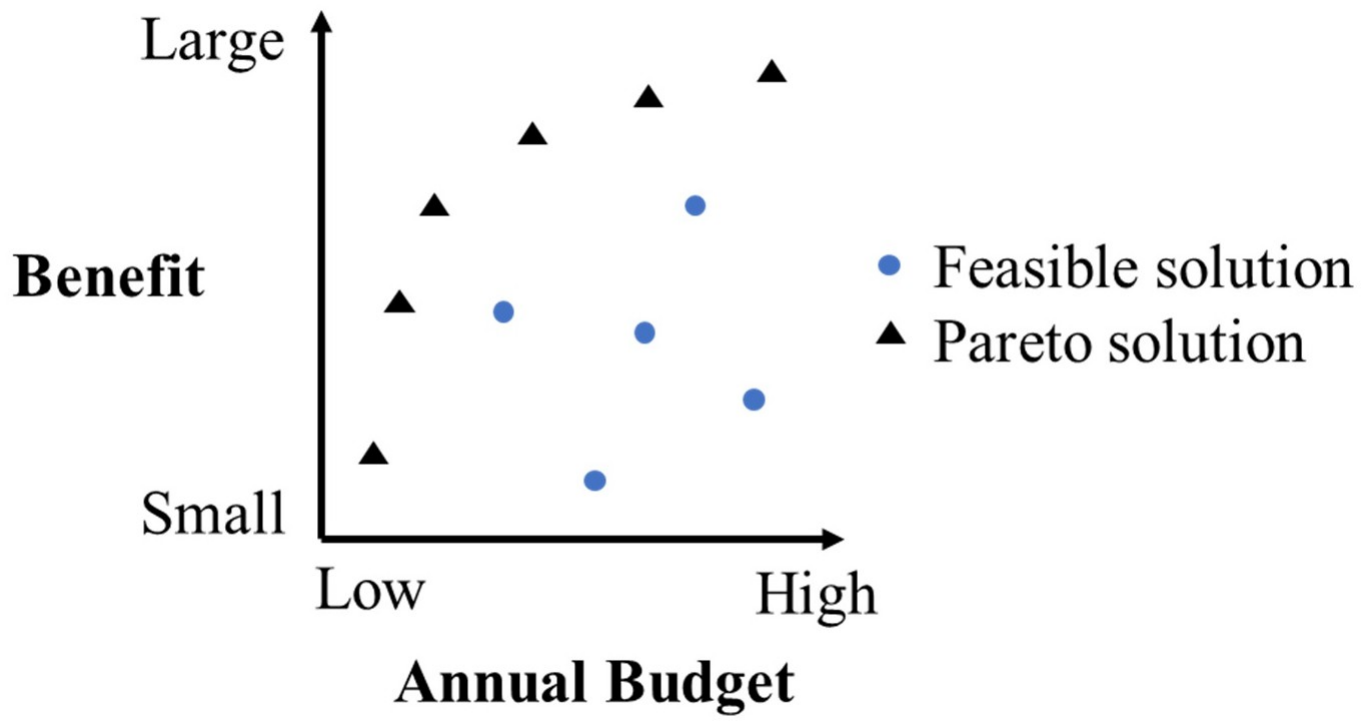
Example of Pareto solutions.

Optimization can analyze M&R outcomes in terms of objectives and constraints when generating M&R strategies. MOO, as a special type of optimization, can simultaneously optimize multiple and even conflicting objectives subject to various constraints, and generate Pareto solutions. The Pareto solution, as demonstrated in Fig 2, refers to a feasible solution, of which any objective value cannot be improved without worsening at least one other objective [45].

**Fig 3:**
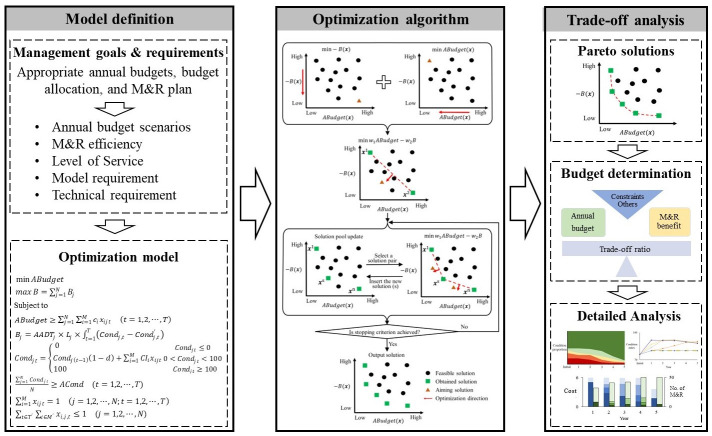
Application framework.

Fig 3 presents the application framework of MOO in M&R decision-making. Based on the goals and requirements of pavement management, decision-making criteria are defined, followed by the establishment of an optimization model. This model has multiple objectives and constraints described by the decision-making criteria. Then an optimization algorithm is applied to generate Pareto solutions. In the context of M&R decision-making, Pareto solutions are the best achievable alternative M&R decisions in a specific situation defined by the objectives and constraints. These solutions do not only contain the best achievable outcomes, but also illustrate the outcome relationship and the consequences when shifting M&R decisions (Pareto solutions). This can be used to balance the annual budgets and other outcomes such as M&R benefits subject to constraints, and therefore reach an informed and compromised M&R decision that best fulfills the M&R targets.

### Decision-making model

This paper assists with decision makers from pavement management departments to enhance the efficiency of M&R decisions by benchmarking the appropriate annual budget and allocating the funding when designing M&R treatments for pavement networks. To achieve this goal, a bi-objective optimization model is established to optimize and trade off annual budgets and M&R efficiency (measured by benefits) on the subject to practical M&R requirements. The formulation of the decision-making model is shown in Eqs (1)–(8), where Eqs (1) and (2) are objectives and others are constraints. The decision variable *x*_*ijt*_ is binary. When *x*_*ijt*_ = 1, treatment *i* is designed for segment *j* in year *t*; otherwise, it is not designed. The detailed explanation of this model is below.

minABudget (1)

$$max\;B=\overset{N}{\underset{j=1}{\sum }}\;B_{j}$$ (2)

$$\begin{array}{cc} \overset{N}{\underset{j=1}{\sum }}\;\overset{M}{\underset{i=1}{\sum }}\;c_{i}x_{ijt}\leq ABudget & \left(t=1,2,\cdots ,T\right) \end{array}$$ (3)

$$\begin{array}{cc} \frac{\sum_{j=1}^{N}\;Cond_{jt}}{N}\geq ACond & \left(t=1,2,\cdots ,T\right) \end{array}$$ (4)

$$Cond_{jt}=\left\{\begin{array}{cc} 0 & Cond_{jt}\leq 0\\ Cond_{j(t-1)}(1-d)+\sum_{i=1}^{M}CI_{i}x_{ijt} & 0<Cond_{jt}<100\\ 100 & Cond_{jt}\geq 100 \end{array}\right.$$ (5)

$$B_{j}=AADT_{j}\times L_{j}\times \overset{T}{\underset{t=1}{\int }}\left(Cond_{j,t}-Cond_{j,t}^{\prime }\right)$$ (6)

$$\begin{array}{cc} \overset{M}{\underset{i=1}{\sum }}\;x_{ijt}=1 & \left(j=1,2,\cdots ,N;t=1,2,\cdots ,T\right) \end{array}$$ (7)

$$\sum_{\begin{array}{l} i\in M^{\prime }\\ t=t^{\prime } \end{array}}x_{ijt}=1\Rightarrow \overset{t^{\prime }+T_{i}^{\prime }}{\underset{t=t^{\prime }}{\sum }}\;\sum_{i\in M^{\prime }}x_{ijt}=1\;\;\left(j=1,2,\cdots ,N;t^{\prime }=1,2,\cdots ,T\right)$$ (8)

where, *ABudget* is the variable of annual budget determined by Eq (3), *B* is the variable of total M&R benefit, *B*_*j*_ is the variable of M&R benefit of segment *j* calculated by Eq (6), *c*_*i*_ is the cost of treatment *i*, *AADT*_*j*_ is AADT of segment *j*, *L*_*j*_ is the lane length of segment *j*, *Cond*_*jt*_ is the condition of segment *j* in year *t* when adopting the designed M&R treatment, $Cond_{jt}^{\prime }$ is the condition of segment *j* in year *t* when no treatment is adopted, *d* is the pavement deterioration rate, *CI*_*i*_ is the condition improvement of treatment *i*, *ACond* is the worst acceptable network condition, *M* is the number of alternative M&R options including no treatment (do-nothing), *M*′ is the set of rehabilitation treatments, *N* is the number of segments, *T* is the number of analysed years, $T_{i}^{\prime }$ is the resting years of rehabilitation treatment *i*.

#### (1) Annual budget

As stated above, annual budgets are often defined as constraints with artificially given values. This is not sufficient for budget analysis. To better understand the variation of annual budgets, this paper defines the annual budget as the objective, namely minimizing the annual budget. The annual budget is defined as the M&R budget in every year during the analysis horizontal, hence, the objective of minimized annual budget is defined as Eq (9).

$$\begin{array}{c} \text{min}\;Budget_{1}\\ \text{min}\;Budget_{2}\\ \vdots \\ \text{min}\;Budget_{T} \end{array}$$ (9)

where, *Budget*_*t*_ is the M&R budget for a network in year *t*.

Often different treatments are designed to different segments, and the required fundings determined by the budgets probably change in different years, i.e. *Budget*_*t*_ ≠ *Budget*_*t*′_ when *t* ≠ *t*′. To ease M&R management, instead of changing values, a constant budget value is often used. In this paper, the annual budget (*ABudget*) presents the constant budget value, which determines the available funding for M&R treatments in every year during the analyzed horizontal. Hence in this paper the annual budget may not be totally spent in a year but cannot be overspent.

To make sure the sufficient funding for all the designed treatments during the analyzed horizontal, *ABudget* is equal to the largest *Budget*_*t*_ (otherwise the budget is not enough for all treatments year *t*). While *Budget*_*t*_ is determined by M&R cost; hence the annual budget (*ABudget*) is also determined by the highest M&R cost in a year *C*^*max*^ during the analyzed period. Thus, the first objective is transformed from minimizing the annual budget to minimizing the highest yearly M&R costs *C*^*max*^ (Eq (10)). In this way, a number of *T* objective functions (Eq (9)) are transformed to one objective function (Eq (1)) and a set of constraints (Eq (3)). This transformation largely reduces the number of objective functions and eases the optimization without affecting the model accuracy. Also, with this definition of the annual budgets *ABudget*, the decision-making model can consider the influence between consecutive years and optimize the budget throughout the entire analysis horizontal.


$$\begin{array}{cc} \text{min}\;ABudget=C^{max}\geq \overset{N}{\underset{j=1}{\sum }}\;\overset{M}{\underset{i=1}{\sum }}\;c_{i}x_{ijt} & \left(t=1,2,\cdots ,T\right) \end{array}$$ (10)

#### (2) M&R benefit

The M&R benefit measures the return on M&R investment, which is an essential criterion measuring the economic efficiency. In this paper, it is defined based on the pavement condition improvement due to M&R treatments. When a M&R treatment is adopted on a pavement segment, the segment condition is improved. The area between the improved condition curve and the condition deterioration curve (no treatment) in Fig 1 is defined as the condition improvement area. M&R benefit of segment *j*, *B*_*j*_, is calculated as the product of Annual Average Daily Traffic (AADT), segment length and condition improvement area using Eq (6). To enhance the investment return, the maximization of the total M&R benefit is defined as the second objective as presented in Eq (2).

#### (3) Pavement condition

The purpose of M&R is to keep pavement infrastructures in acceptable condition, thus, the pavement condition should be considered when making M&R decisions. The pavement condition deteriorates until a M&R treatment is applied as shown in Fig 1. Eq (5) simulates the segment condition variation, where *Cond*_*jt*_ is an integrated condition index ranging between 0 and 100 with 0 presenting the worst and 100 presenting the best. If no treatment is designed in year *t* for segment *j*, its condition index decreases with a deterioration rate of *d* according to its condition in the previous year (*t* − 1). If a treatment *i* is applied, the segment condition index decreases in the year *t* and jumps up at the end of the year.

Because the maximum and minimum value of the condition index is 100 and 0, respectively, the pairwise function Eq (5) ensures that when the calculated condition index considering the decrease due to segment deterioration and the increase due to M&R treatments is larger than 100 or smaller than 0, the variable *Cond*_*jt*_ is set to 100 or 0.

To guarantee a satisfied network and provide the required level of service, the optimization model requires that the average condition index of the network should not be worse than the acceptable value *ACond* across the analysis horizontal as formulated in Eq (4). The purpose of this paper is to trade-off and determine annual budgets, so a linear deterioration model with a constant rate *d* is used to describe the condition deterioration of pavement segments. While other deterioration models can also be used.

#### (4) Model constraint

When planning M&R treatment, a segment can only have one M&R option in a year including no treatment. Eq (7) ensures that exactly one M&R option is designed for each pavement segment in a year.

#### (5) Technical constraint

When designing M&R treatments, the frequency of the rehabilitation treatments is often controlled, because these treatments block the road and heavily affect the traffic for an extended amount of time. Hence, in practice, the rehabilitation treatments cannot be adopted on a segment in consecutive years, and resting time is often needed after rehabilitation. When the resting time of rehabilitation is needed, Eq (8) describes when a rehabilitation treatment is designed to a segment, no other rehabilitation treatment from the rehabilitation set *M*′ will be designed for this segment during the resting time. Therefore, the continuous rehabilitation on a same segment is avoided. It is important to notice that when resting is not needed after rehabilitation, i.e. rehabilitation treatments can be applied on a segment in consecutive years, this constraint (Eq (8)) can be simply removed from the decision-making model and the proposed method and algorithm can also be used.

Eqs (1)–(8) are the formulation of the network-level M&R decision-making. It has two objectives and multiple constraints, which describes the relationship between the minimized annual budget and the maximized M&R benefit subject to the satisfied pavement condition, the model constraint and the technical constraint. This paper uses M&R benefit to measure the decision efficiency, and a bi-objective optimization model is used. However, other criteria can also be used, and multiple objectives can be defined. Then an effective optimization algorithm is needed to optimize the M&R decisions and present the choices for minimized annual budgets and their consequences. Therefore, decision makers can make informed decisions.

### Optimization algorithm

There are two types of MOO algorithms commonly used in M&R decision-making process: heuristics and exact methods. Heuristics are popular choices because they are fast and robust [41]. Yet as the randomness in the nature, they cannot guarantee to generate the real Pareto solutions. Exact methods based on mathematical theories often have the deterministic procedures, and their solutions quality are guaranteed. With the help of programming tools, exact methods can fast analyze MOO problems and obtain real Pareto solutions. In addition, heuristics may need parameter calibration or a significant amount of training data [46–48], which could be difficult for M&R decision-making especially when decision makers do not have enough knowledge about the addressed problems. Compared to heuristics, exact methods are easier for applications. Hence, in this paper, an exact MOO method named dichotomic approach is introduced.

**Fig 4:**
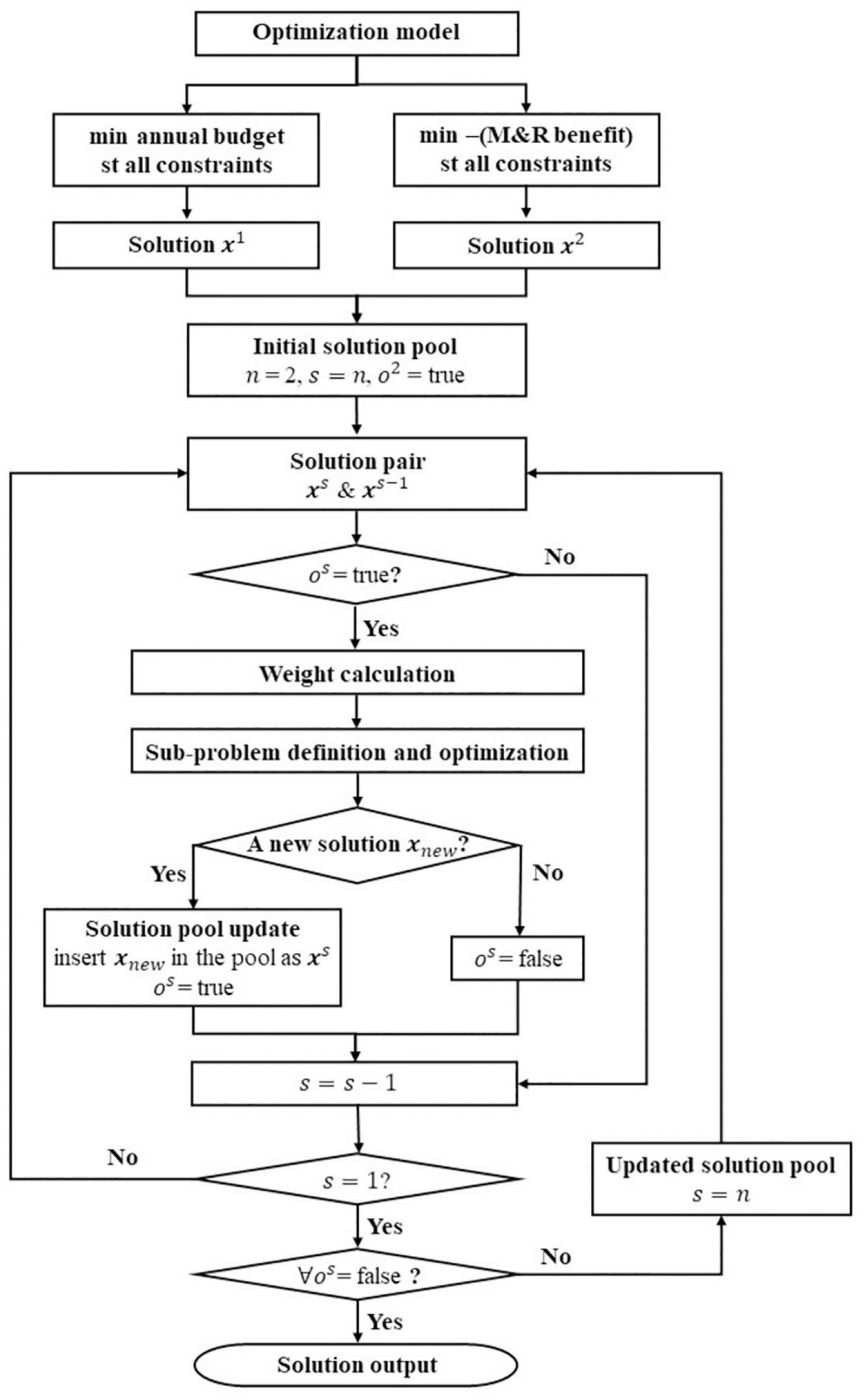
Algorithm of dichotomic approach.

The dichotomic approach dynamically transfers a MOO problem into Single-Objective Optimization (SOO) sub-problems in an iterative manner and solves the sub-problems to generate solutions. Compared to heuristics, it guarantee the solution quality. Compared to other exact methods, it is fast and efficient [49]. Fig 4 demonstrates the algorithm of the dichotomic approach.

Step 1: Initialization. Firstly, two SOO sub-problems are generated by optimizing one of the two objectives subject to all the constraints. To ease the calculation, the objective max *B* is transformed into min −*B*. These two sub-problems are separately solved by the branch and bound method [50], each leading to an optimal solution (two in total). The two solutions (***x***^1^ and ***x***^2^) are added into an empty solution pool, where *n* is the number of solutions in the pool, *s* is the sequence of solution ***x***^*s*^ in the pool. The Boolean variable *o*^*s*^ with an initial value of true presents whether the solution ***x***^*s*^ and its consecutive solution ***x***^*s*−1^ need to be analyzed. Due to the definition, *o*^1^ does not exist.

Step 2: Solution selection and weight calculation. For a pair of consecutive solutions (***x***^*s*^ and ***x***^*s*−1^) with true *o*^*s*^, two weights (*w*_1_ and *w*_2_) are calculated by Eqs (11) and (12), where *B*(***x***) and *ABudget*(***x***) are the benefit and the annual budget of solution ***x***. If the variable *o*^*s*^ is false, the algorithm moves to the next solution pair (*s* = *s* − 1).


$$w_{1}=B\left(x^{s-1}\right)-B\left(x^{s}\right)$$ (11)

$$w_{2}=ABudget\left(x^{s-1}\right)-ABudget\left(x^{s}\right)$$ (12)

**Fig 5:**
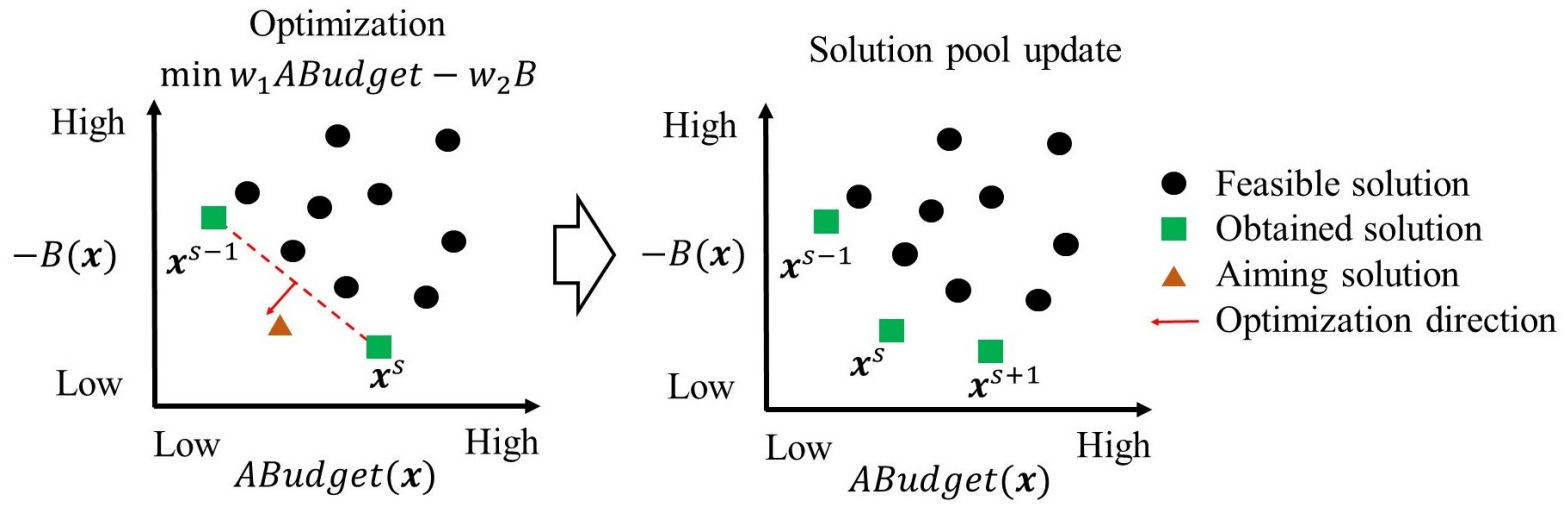
Example of the solution generation.

Step 3: Sub-problem definition and optimization. With the weights obtained in Step 2, a new objective is defined by weighted summing the objectives using Eq (13). Then a sub-problem is defined by optimizing the new objective subject to all the original constraints in Eqs (3)–(8). This sub-problem is solved with the branch and bound method. As shown in Fig 5, if an optimal solution ***x***_*new*_ is generated and not in the solution pool, it is inserted into the pool with the sequence of *s* and the Boolean variable *o*^*s*^ of true; otherwise, the Boolean variable *o*^*s*^ turns to false, and the algorithm moves to the next solution pair (*s* = *s* − 1). The solution sequence is updated accordingly.


$$\text{min}\;w_{1}ABudget-w_{2}B$$ (13)

Step 4: Iteration check. When the sequence *s* = 1, which means all the consecutive solution pairs in the pool are analyzed, the algorithm goes to Step 5; otherwise, it returns to Step 2.

Step 5: Termination check. If all the Boolean variable *o*^*s*^ is false, which indicates all solution pairs are analyzed and no new solution is found in the last iteration, this algorithm stops and outputs the solutions in the pool; otherwise, it goes back to Step 2.

The dichotomic approach iteratively generates a set of Pareto solutions in an efficient way. These solutions are the optimized decisions of M&R plans with different annual budgets and M&R benefits subject to all the constraints, which can help determining an informed annual budget.

### Trade-off analysis

Trade-off analysis balances the desirable but incompatible M&R outcomes in terms of multiple objectives by executing different M&R decisions for a pavement network during a finite planning horizon [51]. It informs decisions to sacrifice optimality in one objective for higher gains in the other objective(s) to better fulfill the M&R targets. Therefore, it helps to quantify and compromise the consequences of different annual budgets with the Pareto solutions.

Pareto solutions represent the unbeatable alternatives of M&R decisions measured by the defined model. They do not only present the lowest annual budget to obtain an acceptable network and the highest achievable M&R benefit, but also illustrate the relationship between the optimized outcomes i.e. minimized annual budget and maximized M&R benefit. When changing a unit of the annual budget, the corresponding return on the M&R benefit is qualified. Furthermore, the optimal consequences when applying different annual budget scenarios are obtained with Pareto solutions. This can be used for budget-shifting analysis, i.e. assessing the M&R benefits when shifting the annual budgets.

This paper introduces the trade-off ratio to assist with the trade-offs between the M&R outcomes. The ratio defined by Eq (14) measures the efficiency of the decision. When the trade-off ratio is higher, more benefit is yielded by increasing a unit of the annual budget, which contributes to more return on the M&R investment. Then decision makers can reach an efficient annual budget and the corresponding M&R decision with the highest trade-off ratio considering the financial pressure and allowance.


$$r=\frac{B\left(x^{s+1}\right)-B\left(x^{s}\right)}{ABudget\left(x^{s+1}\right)-ABudget\left(x^{s}\right)}$$ (14)

## Case study

### Case information

The proposed method is applied to a pavement network of 50 pavement segments in Texas, and M&R decisions are taken over an analysis period of five years as an example. The pavement attributes were retrieved from the Department of Transportation in Texas (TxDOT). Table 1 summarizes the statistical description of the salient attributes of the network, where the original data is provided as S1 Data. The analyzed segments were hot-mix asphalt with a length of 0.1 to 0.6 miles and 2 to 4 lanes.

**Table 1:** Statistic summary of the analyzed pavement network.

Attribute	Mean	Minimum	Maximum	Standard Deviation
Condition*	78.90	8	100	24.61
AADT	7850.80	540	23000	6814.99

* An integrated condition index in the initial year.

### Implementation

TxDOT has five primary M&R options: no treatment, preventive maintenance, light rehabilitation, medium rehabilitation, and heavy rehabilitation. The information is summarized in Table 2. For the purpose of demonstrating the proposed method, the assumed values are used in the case study, including the worst acceptable network condition *ACond* = 80, and the deterioration rate *d* = 5%. It is assumed that at most only one rehabilitation treatment can be applied to a segment in five years. The case is programmed using Python 3.7.

**Table 2:** Summary of the potential M&R treatments*.

M&R	Treatment cost ($ thousand/lane-mile)	Condition improvement
No treatment	0	0
Preventive maintenance	34	3
Light rehabilitation	202	15
Medium rehabilitation	277	25
Heavy rehabilitation	517	40

*Source: Based on France-Mensah & O’Brien [52].

## Result and discussion

**Fig 6:**
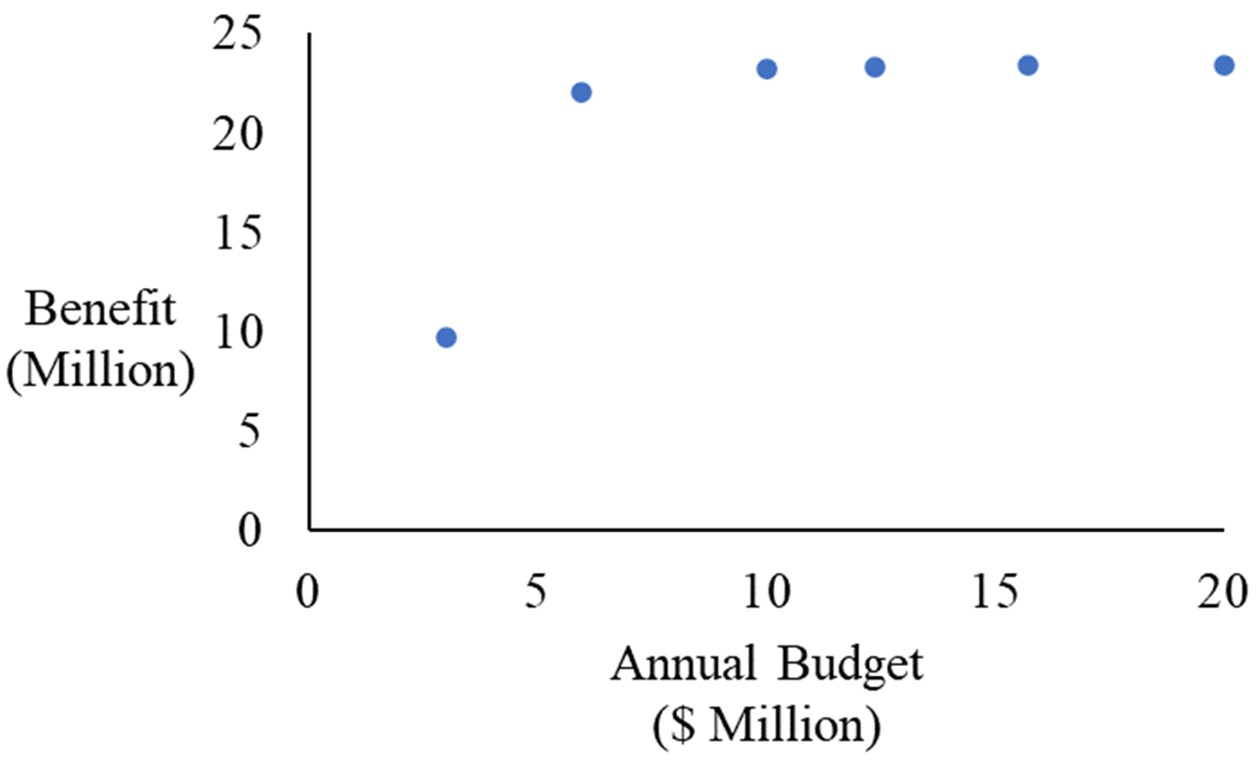
Generated Pareto solutions.

The case study is modeled and analyzed with the developed method, and six Pareto solutions are generated. These solutions are presented in Fig 6 and Table 3. Each solution is an unbeatable M&R decision that satisfies all the constraints and achieves the objectives in the best possible manner measured by the optimization model of Eqs (1)–(8).

**Table 3:** Generated Pareto solutions and their trade-off ratios.

Solution Index	Annual budget ($ Million)	M&R benefit (Million)	Trade-off ratio
A	2.9945	9.7189	
B	5.9575	22.0047	4.1464
C	9.9991	23.1756	0.2897
D	12.3936	23.3366	0.0672
E	15.7188	23.3866	0.0150
F	20.0000	23.3877	0.0003

**Fig 7:**
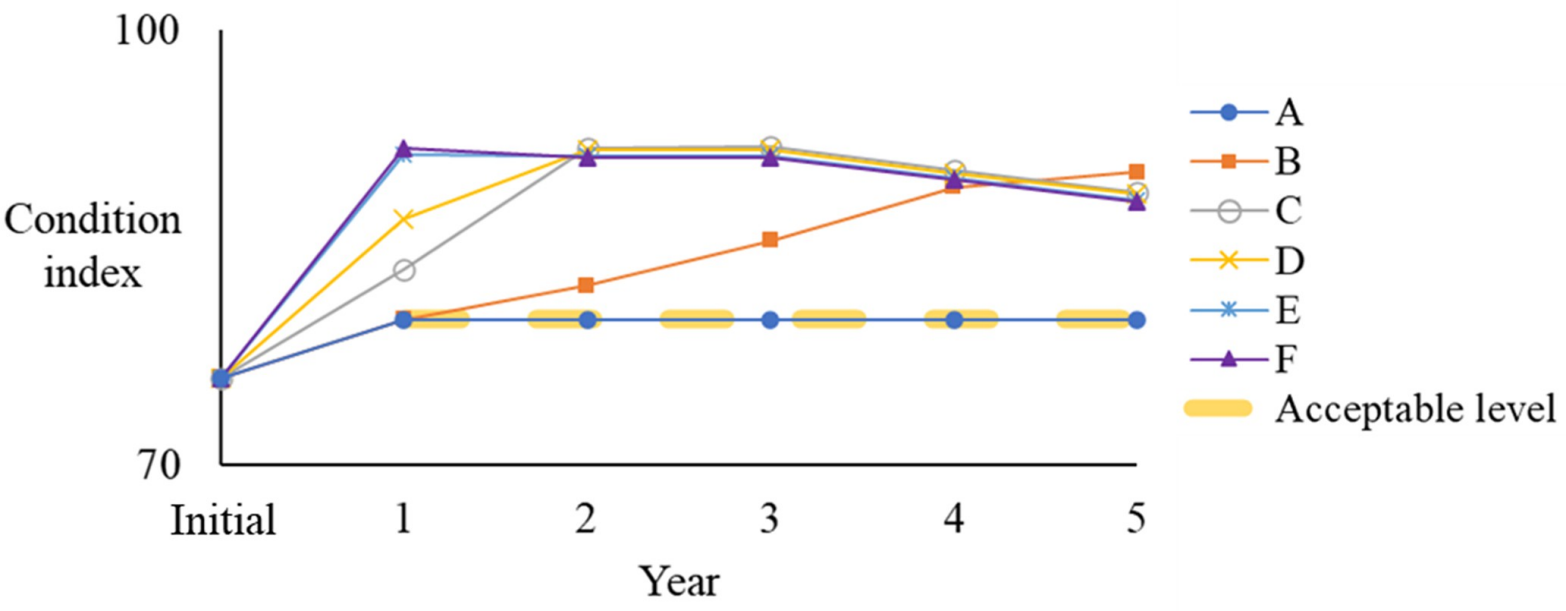
Condition variation.

In this case, the least annual budget of $2.9945 million is required to maintain an acceptable network during the analysis horizontal (Solution A). Fig 7 presents the condition variation of the solutions. Due to the limited funding, Solution A only allows the cheap and necessary M&R treatments, and its condition merely reaches the requirement, i.e. average network condition is not worse than 80.

Compared to Solution A, Solution F is the opposite. It requires an annual budget of $20 million for M&R treatments and achieves the highest M&R benefit of 23.3877 million. Therefore, even if the funding is sufficient, the annual budget should not exceed $20 million for this network. In this paper, the M&R benefit is measured by Eq (4), which indicates earlier treatment generates higher benefits. Thus, as demonstrated in Fig 7, even the condition of Solution F is not always better than the condition of Solutions B and C, its benefit is higher as the annual budget of Solution F allows more treatments at an early stage, which contributes to the higher benefit. It is important to observe that in this case, a segment can only have one rehabilitation treatment during the analysis horizontal, hence, the condition cannot be kept at 100 considering the pavement deterioration.

**Fig 8:**
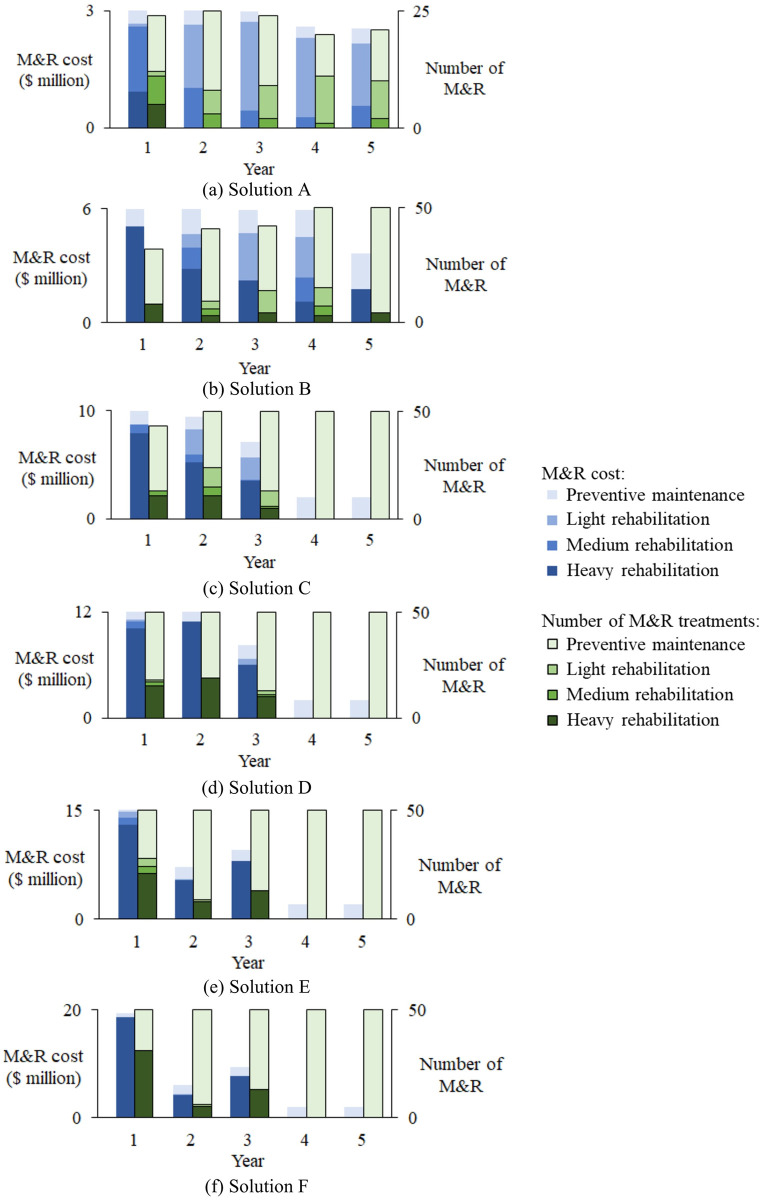
Budget allocation and M&R design.

Fig 8 illustrates the budget allocation and treatment numbers. When the annual budget increases from Solutions A to F, more segments have funding for heavy rehabilitation at the early stage and therefore achieve more benefits. More specifically, when the budget is low, expensive treatments are limited, and major funding is spent on the preventive maintenance and light rehabilitation. These treatments require a small amount of money and slightly improve the pavement condition. Moreover, the number of treated segments is limited due to insufficient funding.

When the annual budget increases, more funding is available and more expensive treatments can be planned. When the annual budget grows to $5.9575 million (Solution B), all segments can be treated in Years 4 and 5, and more funding is allocated to medium and heavy rehabilitations that are expensive but obviously improve the pavement condition. When the annual budget keeps increasing, the major funding is allocated to heavy rehabilitation in the early stage and therefore generate high benefit. In this case, only one rehabilitation treatment can be designed for a segment during the analysis horizontal. Hence the sufficient funding allows all segments to be rehabilitated in the first three years, and only preventive maintenance is needed in Years 4 and 5. Therefore only a little funding is needed in Years 4 and 5.

In general, when the annual budget increases, the M&R benefit is also growing, while the increasing ratio is different. As depicted in Table 3 when the annual budget increases from $2.9945 million to $5.9575 million, the M&R benefit reaches 22.0047 million with the largest trade-off ratio of 4.1464, which indicates with one extra dollar on the annual budget, the benefit of 4.1464 can be returned. Then the trade-off ratio decreases with the increasing annual budget, which implies the return on the benefit is reducing. Hence, in this case, to make the most effective use of the funding, the M&R decision could be set as Solution B with the annual budget of $5.9575 million and the M&R benefit of 22.0047 million.

As shown in Fig 8, the proposed method also considers the influence between the consecutive years. M&R decisions (solutions) are generated at comprehensive level, where treatments are planned for all segments throughout the entire horizontal. For example, Solution B spends most funding on the heavy rehabilitation and therefore generates high benefit in the first year. As the heavy rehabilitation is extremely expensive, only 32 segments can be treated in the first year. Due to the pavement deterioration, the preventive maintenance can only reduce the deterioration speed. To improve the network condition and enhance the benefit, the large proportion of the funding is allocated to the rehabilitation treatments in the first four years as shown in Fig 8. In addition, from Year 1 to 4, funding on heavy rehabilitation is decreasing, and increasing funding is spent on medium and light rehabilitations to ensure that the good segments (*Cond* > 60) can be treated effectively.

In the final year, because of the rehabilitation limitations (one rehabilitation for a segment), only four segments can be rehabilitated, and the other 46 segments are designed with cheap and unlimited preventive maintenance. This is the reason why only 61 percent of the annual budget is used in the final year. When the rehabilitation limitation is not needed, more rehabilitations would be designed, and more funding would be spent.

According to the case study, the proposed method can be used to determine the appropriate annual budget and design the M&R treatments for pavement networks. It does not only generate the Pareto solutions representing the best M&R decisions, but also provides the best alternative choices of the annual budgets and their consequences when shifting the optimized M&R decisions. With the provided information, the decision makers are able to know and balance the annual budgets and their consequences, therefore achieve an appropriate decision. The proposed method can also be applied to the long-term M&R planning and large networks, and the algorithm may need an extensive amount of time to generate all the Pareto solutions.

## Conclusion

This paper discusses the network-level pavement M&R decision-making with an optimized annual budget. It studies the determination of the annual budgets when planning M&R treatments for pavement networks, and therefore enhances the economic efficiency of M&R. The proposed method is applied to a pavement network in Texas. Conclusions are drawn as follows:

1. Different from the previous research where the annual budgets are given values, a MOO based method is developed to help determining the annual budgets and allocating the available funding by generating optimized M&R plans.
2. This method optimizes and trades off the annual budgets and their consequences subject to the constraints. The feasibility of the developed method is examined by a pavement network of 50 segments in Texas. 6 alternative strategies with the minimized annual budgets and the maximized benefits are obtained, which can help determining the appropriate annual budget level and the corresponding funding allocation.
3. The proposed method is proven to be effective when analyzing a pavement network with 50 segments for 5-year analysis. Long-term M&R planning and large networks can also be analyzed, while the algorithm may need an extensive amount of time.
4. The proposed method can help decision-makers to understand the pavement networks and achieve informed M&R decisions. It can assist with the pavement management and improve its economy and financial situation.

This paper develops a method that does not only allocate the available funding and design the M&R treatments, but also trades off the choices of optimized annual budgets. When other criteria need to be balanced in terms of objectives, the decision-making model is modified, and the efficiency of the optimization algorithm should be further discussed.

## Supporting information

S1 Data Data of the studied case. (XLS)
